# Controlled drug delivery vehicles for cancer treatment and their performance

**DOI:** 10.1038/s41392-017-0004-3

**Published:** 2018-03-16

**Authors:** Sudipta Senapati, Arun Kumar Mahanta, Sunil Kumar, Pralay Maiti

**Affiliations:** School of Materials Science and Technology, Indian Institute of Technology (Banaras Hindu University), Varanasi, India

## Abstract

Although conventional chemotherapy has been successful to some extent, the main drawbacks of chemotherapy are its poor bioavailability, high-dose requirements, adverse side effects, low therapeutic indices, development of multiple drug resistance, and non-specific targeting. The main aim in the development of drug delivery vehicles is to successfully address these delivery-related problems and carry drugs to the desired sites of therapeutic action while reducing adverse side effects. In this review, we will discuss the different types of materials used as delivery vehicles for chemotherapeutic agents and their structural characteristics that improve the therapeutic efficacy of their drugs and will describe recent scientific advances in the area of chemotherapy, emphasizing challenges in cancer treatments.

## Cancer therapeutics: Special delivery

Improving the delivery of cancer therapies to tumor sites is crucial to reduce unwanted side effects and patient mortality rates. Pralay Maiti and colleagues at the Indian Institute of Technology in Varanasi, India, review the latest developments in drug delivery vehicles and treatment approaches designed to enhance the effectiveness of current cancer therapies. New nanoparticle-based carriers, hydrogels and hybrid materials that offer controlled and sustained drug release are showing great promise in animal models. Furthermore, materials that respond to stimuli such as heat, light, magnetic or electric fields are also being tested to aid target-specific drug delivery and, thus, avoid damage to healthy tissues. Although there are some challenges in translating these findings to the clinic, there is no doubt that technological advances are shaping better and safer treatment options.

## Introduction

Cancer includes a range of diseases that arise as a result of the unregulated growth of malignant cells, which have the potential to invade or spread to other body parts. With more than 10 million new cases each year, cancer-related deaths are projected to increase in the near future with an estimation by the World Health Organization of ~13.1 million cancer-related deaths by the year 2030^1^. However, the mortality rate has decreased in the past 5 years due to a better understanding of tumor biology and improved diagnostic devices and treatments. Current cancer treatment options include surgical intervention, chemotherapy, and radiation therapy or a combination of these options. Conventional chemotherapy works primarily by interfering with DNA synthesis and mitosis, leading to the death of rapidly growing and dividing cancer cells. The agents are nonselective and can also damage healthy normal tissues, causing severe unintended and undesirable side effects, e.g., loss of appetite and nausea. In fact, the severe adverse effects induced by chemotherapeutic drugs on healthy tissues and organs are a major reason behind the high mortality rate of cancer patients. Additionally, as the bio-accessibility of these drugs to tumor tissues is relatively poor, higher doses are required, leading to elevated toxicity in normal cells and an increased incidence of multiple drug resistance. Therefore, it is desirable to develop chemotherapeutics that can either passively or actively target cancerous cells, thereby reducing adverse side effects while improving therapeutic efficacy. In the last few years, a better understanding of tumor biology and increased availability of versatile materials, including polymers^2–5^, lipids^6,7^, inorganic carriers^8^, polymeric hydrogels^9,10^, and biomacromolecular scaffolds^11^, have led to the development of systems that can deliver chemotherapeutics to tumor sites with improved therapeutic efficacy. The emergence of nanotechnology has had a profound impact on clinical therapeutics in general in last two decades. Compared to conventional chemotherapeutic agents, nanoscale drug carriers have demonstrated the potential to address some of these challenges by improving treatment efficacy while avoiding toxicity in normal cells due to features such as high selective accumulation in tumors via the enhanced permeability and retention (EPR) effect and active cellular uptake^12,13^. Active targeting approaches may be achieved by conjugating nanocarriers containing chemotherapeutics to molecules that bind to over expressed antigens. Among emergent nanoscale drug carriers, liposomes, polymeric nanoparticles, and micelles have demonstrated great potential clinical impacts. At present, several nanoparticle-based chemotherapeutics are clinically approved and many more are in various stages of clinical or preclinical development. Although nanocarriers offer many advantages as drug carrier systems, their lack of biodegradation, poor bioavailability, instability in the circulation, inadequate tissue distribution and potential toxicity raise concerns over their safety, especially for long-term administration. Cancer chemoresistance, which is accountable for most failure cases in cancer therapy, is a phenomenon in which cancer cells that are initially suppressed by an anticancer drug develop resistance towards the particular drug. For this reason, novel drug delivery systems with better targeting ability are needed for cancer prevention, the suppression of adverse side effects and pain management associated with cancer chemotherapy.

In this review article, we discuss various drug delivery vehicles used in cancer therapeutics to increase the therapeutic index of chemotherapeutic drugs. The performance of basic research to clinical studies in the context of present day oncological development is discussed. This review presents current challenges associated with chemotherapy, followed by a discussion about the future directions of chemotherapy.

## Materials and strategies used in cancer therapy

**Scheme 1:**
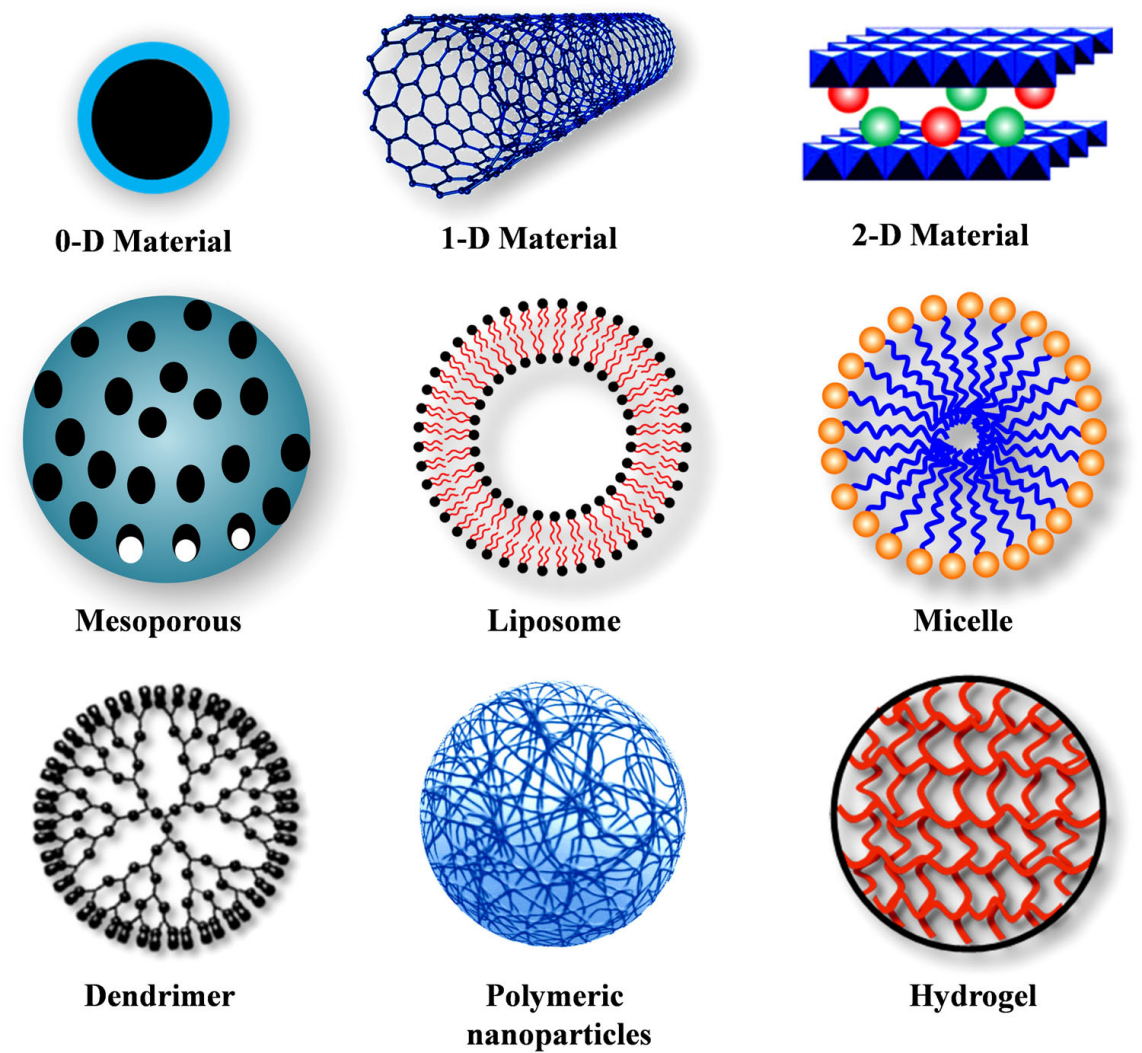
Different types of nanocarriers used as controlled delivery vehicles for cancer treatment

Several innovative methods of drug delivery are being used in cancer treatment. A wide range of nanoscale compounds based on synthetic polymers, proteins, lipids, and organic and inorganic particles have been employed for the development of cancer therapeutics. Compared with the direct administration of bare chemo-drugs, drug encapsulation in a carrier offers a number of advantages, such as protection from degradation in the bloodstream, better drug solubility, enhanced drug stability, targeted drug delivery, decreased toxic side effects and improved pharmacokinetic and pharmacodynamic drug properties. To date, an impressive library of various drug delivery vehicles has been developed with varying sizes, architectures, and surface physicochemical properties with targeting strategies (Scheme 1). Table 1 summarizes some examples of drug delivery systems that have either been approved or are in clinical or preclinical development stages.

**Table 1:** Various drug delivery carriers used in cancer therapy

Material	Description of Carrier	Commercial Name	Material Advantage	Specificity	Ref
Carbon nanotube	Anti-P-glycoprotein antibody functionalized CNT-doxorubicin		Overcomes multidrug resistance	Human leukemia cells (K562)	191
Layered double hydroxide (LDH)	Co-delivery of 5-fluorouracil and siRNAs		Overcomes drug resistance and enhances cancer treatment	Tested on three different cancer cell lines	38
	Raloxifene intercalated into the interlayer gallery of LDH host		Enhanced therapeutic efficacy; reduction of adverse side effects, pure drug	Solid tumor	33
Iron oxide nanoparticles	Phospholipid-PEG coated superparamagnetic iron oxide nanoparticles	Nano Therm	Both chemotherapy and hyperthermia treatment	Solid cancer	192
Mesoporous silica nanoparticles (MSN)	Azobenzene-modified mesoporous silica for NIR-triggered anticancer drug delivery		Drug release rate can be controlled by varying the intensity and/or time	Solid tumor	193
	Endosomal pH-sensitive MSN for doxorubicin delivery		Enhances chemotherapeutic efficacy and overcomes multidrug resistance	Solid tumor	194
Polymeric nanoparticles	Cyclodextrin-PEG nanoparticles covalently conjugated with camptothecin	CRLX101	Specifically delivers anticancer agents to tumor tissues and reduces side effects	Lung and ovarian cancer	195
	PEG-PLGA nanoparticle formulation of docetaxel	BIND-014	Controlled biodistribution, targeted and preferential tumor accumulation result in increased efficacy and decreased toxicity	Various solid malignancies	196
Liposomes	Liposomal doxorubicin	Doxil	Improved delivery to site of disease; decrease in systemic toxicity of free drug	Karposi’s sarcoma; Ovarian cancer; multiple myeloma	197
	Liposomal cytarabine	Myocet	Increased delivery to tumor site; lower systemic toxicity arising from side effects	Intrathecal lymphomatous meningitis	198
	Liposomal daunorubicin	DaunoXome	Enhanced delivery to tumor site; lower systemic toxicity arising from side effects	Karposi’s sarcoma	199
Micelle	Polymeric methoxy-PEG-poly(D,L-lactide) micelle formulation of paclitaxel	Genexol-PM	Improved delivery to site of disease; decrease in systemic toxicity of free drug.	Breast cancer; lung cancer; ovarian cancer	200
	PEG-b-poly(α,β-aspartic acid) nanoparticle formulation of paclitaxel	NK 105	Enhanced antitumor efficacy and dramatically lower neurotoxicity than free paclitaxel	Gastric cancer; breast cancer	201
Protein nanoparticles	Human serum albumin-bound paclitaxel nanoparticles	Abraxane	Improved solubility; improved delivery to tumor	Metastatic breast cancer	202
	Folate-conjugated bovine serum albumin-bound paclitaxel nanoparticles		Increased solubility, cellular uptake; targeted specifically to cancer cells	Human prostate cancer cells (PC3)	82
Dendrimer	Carboxylated PAMAM dendrimers covalently conjugated with cisplatin		Improved loading efficiency, reduced cytotoxicity; significant anti-proliferative activity against lung cancer	Lung cancer cells (NCI-H460)	203
	Complexation of doxorubicin with cationic poly-L-lysine dendrimer		Significant increment in therapeutic efficacy of the drug upon complexation both in vitro and in vivo	Solid tumor	204

### Nanocarriers for drug delivery

Nanomedicine is a rapidly developing area that is revolutionizing cancer diagnosis and therapy. Nanoparticles have unique biological properties given their small size (diameter within 1–100 nm) and large surface area to volume ratio, which allows them to bind, absorb and carryanticancer agents, such as drugs, DNA, RNA, and proteins, along with imaging agents with high efficiency. Nanocarriers used in chemotherapy can be classified into two major types designed for targeted or non-targeted drug delivery: vehicles that use organic molecules as a major building block material and those that use inorganic elements (usually metals) as a core. Organic nanocarriers are comprised of liposomes, lipids, dendrimers, carbon nanotubes, emulsions, and synthetic polymers.

#### Inorganic nanocarriers

Inorganic nanocarrier platforms have been intensively investigated for therapeutic and imaging treatments in recent years due to their great advantages, such as large surface area, better drug loading capacity, better bioavailability, lower toxic side effects and controlled drug release, and their tolerance towards most organic solvents, unlike polymer-based nanoparticles. Quantum dots, carbon nanotubes, layered double hydroxides, mesoporous silica and magnetic nanoparticles are commonly used in cancer treatment in various ways. Quantum dots have already been proven to be powerful imaging probes, especially for long-term, multiplexed and quantitative imaging and diagnostics^14–16^. Zero dimensional (0-D) fluorescent nanoparticles, such as quantum dots (QDs) within the size of 1–10 nm, have emerged as one of the most promising nanoparticles for targeted and traceable drug delivery systems, real-time monitoring of intracellular processes and in vivo molecular imaging due to their unique physicochemical properties, such as uniform size, large surface-to-volume ratio, biocompatibility, highly tunable photoluminescence property, improved signal brightness, resistance against photobleaching and multi-color fluorescence imaging and detection^17^. However, the main challenge with QDs in biological applications is their hydrophobic nature, high tendency of aggregation and non-specific adsorption^18,19^. QD surfaces are usually coated with polar species and/or monolayer or multilayer ligand shells to make them water soluble and to enhance their bioactivity^20^. This type of coating also helps in the development of multifunctional QDs, where imaging contrast agents and small molecular hydrophobic drugs can be embedded between the inorganic core and the amphiphilic polymer coating layer while hydrophilic therapeutic agents (hydrophilic drug, small interfering RNA (siRNA), etc.) and targeting biomolecules (antibodies, proteins, peptides, and aptamers) can be immobilized onto the hydrophilic side of the amphiphiles^21,22^. Gao *et al.*^23^ developed polymer encapsulated and bioconjugated QD probes for cancer targeting and in vivo imaging. d-α-Tocopheryl polyethylene glycol 1000 succinate mono-ester (TPGS) coated multifunctional (theranostic) liposomes have been developed in the form of docetaxel and QD for cancer-imaging and targeted therapy^24^. Recently, multifunctional QDs have been synthesized, making them a promising targeted drug delivery vehicle for the diagnosis and image-guided chemotherapy of various cancers^25,26^.

Carbon nanotubes (CNTs) are synthetic one-dimensional (1D) nanomaterials made from carbon, and they structurally contain rolled sheets of graphene rings built from *sp*^2^ hybridized carbon atoms into hollow tubes. CNTs are well known for ideal near-infrared photothermal ablation therapy because they increase the temperature within tumors as a function of light intensity and CNT dose^27,28^. Functionalized water-soluble CNTs are being investigated for their use in gene and drug delivery because they can readily cross biological barriers and can effectively transport molecules into the cytoplasm without producing a toxic effect^29,30^. Chemotherapeutic drug molecules have been conjugated to functional groups on the CNT surface or through polymer coatings of CNTs, which are usually formed via cleavable bonds. CNTs for antitumor immunotherapy can act as antigen-presenting carriers to improve weakly immunogenic tumor-based peptides/antigens to trigger a humeral immune response within the tumor^31,32^.

**Fig. 1:**
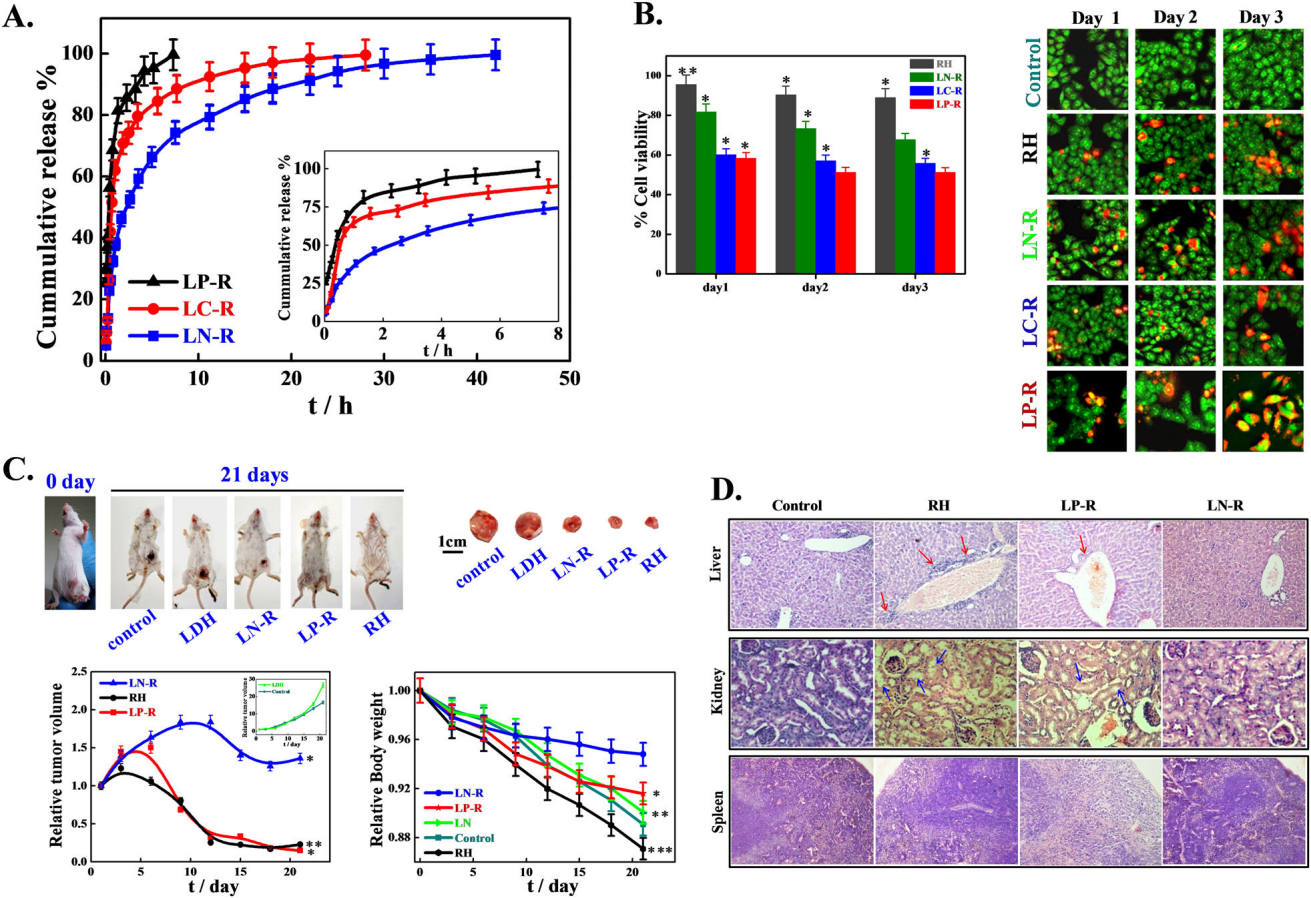
In vitro and in vivo controlled release of drug using layered double hydroxides and its effects. **a** In vitro drug release profiles for drug intercalated nitrate, carbonate and phosphate LDHs (LN-R, LC-R and LP-R, respectively); inset figure describes the release pattern of the above mentioned systems in a time frame of 0–8 h; **b** In vitro cytotoxicity of free drug and drug intercalated LDHs against HeLa cells at different time intervals; **c** In vivo antitumor effect and systematic toxicity of pure RH and drug intercalated LDHs in comparison to control; and **d** Histological analysis of liver, kidney and spleen of tumor bearing mice treated with control (saline), pure RH, LN-R and LP-R^33^

Among the inorganic nanocarriers, two-dimensional (2D) layered double hydroxides (LDHs), also known as hydrotalcite-like compounds, have recently attracted a great deal of interest for their potential as delivery carriers mainly because of their excellent biocompatibility, anion exchange capability, high drug loading efficacy, full protection for loaded drugs, pH-responsive drug release, ease of preparation, low cost, easy, and efficient penetration into the cell membrane and considerable drug delivery, biodegradation in the cellular cytoplasm (pH between 4 and 6), and good endosomal escape; moreover, the drug release rate can be tuned by changing the interlayer anion. LDHs consist of layers of a divalent metal ion, such as Mg^2+^, Ca^2+^, Ni^2+^, and Zn^2+^, with a trivalent metal ion isomorphically substituted to give the layers a net positive charge^33^. This charge is balanced by interlayer hydrated anions, resulting in a multilayer of alternating host layers with exchangeable gallery anions, such as Cl^−^, NO_3_^−^, and CO_3_^2−^. Anionic drugs and biofunctional molecules (genetic materials, peptides, proteins, etc.) can easily be intercalated in the interlayer gallery through direct synthesis, coprecipitation, anion exchange, etc., thereby conferring protection against enzymatic degradation while flowing in biological fluids^34–36^. In addition, their internal and/or external surfaces can easily be functionalized and modified to incorporate a targeting function, and their high specific surface area and better chemical stability make them attractive for diverse applications. LDHs can intercalate various important anionic biofunctional molecules, such as DNA, siRNA, nucleotides and anticancer drugs, showing sustained delivery with high therapeutic efficiency and bioactivity. A unique strategy for the delivery of non-ionic insoluble drugs using LDH as carrier can also be made through micellization^37^. The intercalation of an anticancer drug, raloxifene hydrochloride (RH), into a series of magnesium aluminum LDHs with varying interlayer exchangeable anions (NO_3_^−^, CO_3_^2−^, and PO_4_^3−^) through an ion exchange technique has been reported and was found to release the drug in a controlled manner^33^. Figure 1a illustrates the rapid release rate using phosphate bound LDH-drug (LP-R) while sustained delivery is obtained using nitrate based LDH (LN-R). Spectroscopic (XPS, UV–vis) and thermal studies (DSC)studies confirm the strong interactions between drug molecules and LDH host layers, which lead to sluggish delivery in LN-R against LP-R. In vitro anticancer studies demonstrate better efficacy of cell death using drug intercalated LDHs instead of a pure drug arising from sustained release of the intercalated drug (Fig. 1b). Among the drug intercalated LDHs, LP-R/pure drug exhibits better tumor suppression efficiency, whereas body weight loss index suggests organ damage. In contrast, LN-R shows slight, slow tumor healing but exhibits minimum body weight loss, indicating a better drug delivery vehicle (Fig. 1c). Histograms of different organs and analyses of biochemical parameters suggest damaged liver cells of mice treated with fast release vehicle (pure drug and LP-R), whereas no damage occurs in mice liver cells treated with LN-R or slow release vehicle (Fig. 1d). Further, positively charged LDH nanoparticles can easily penetrate into negatively charged cell membranes through the clathrin-mediated endocytosis pathway. Li *et al.*^38^ employed a combined strategy using LDH to simultaneously deliver CD-siRNA and a chemotherapeutic drug (5-fluorouracil; 5-FU) to cancer cells, leading to significantly higher cytotoxicity than single treatments with either CD-siRNA or 5-FU.

Fullerenes are carbon allotropes with a large spheroidal molecule consisting of a hollow cage of sixty or more atoms. They behave like electron deficient alkenes and react readily with electron rich species^39^. The photodynamic effect of two new decacationic fullerene and red light-harvesting antenna-fullerene conjugated monoadduct derivatives generated reactive oxygen species (ROS) for anticancer therapy. Mesoporous silica nanoparticles (MSNs) are extensively used as drug delivery vehicles due to their unique properties, such as their large specific surface area and pore volume, controllable particle size, ease of functionalizing good biocompatibility and ability to provide a physical casing to protect and house drugs from degeneration or denaturation. MSNs with tunable pore sizes offer great potential for controlling drug loading percentages and release kinetics and can deliver antitumor drugs in a targeted fashion, releasing them on demand to increase their cellular uptake without any premature release prior to reaching the target site^40^. Another advantage of MSNs is their ability to deliver membrane impermeable hydrophobic drugs, thereby serving as a universal transmembrane carrier for intracellular drug delivery and imaging applications. They also have emerged as promising candidates for both passive and active targeted delivery systems and can accumulate in tumor tissues via the enhanced permeation and retention (EPR) effect. Furthermore, specific drug delivery can be achieved via active targeting by the functionalizing of MSNs with targeting ligands, such as folate (FA) or EGF^41^. Antibodies, peptides, and magnetic nanoparticles can also be decorated onto MSNs, thereby acting as a homing device. In the targeting process, particle size and surface modification of MSNs critically influence particle cellular uptake, pharmacokinetics, and biodistribution profiles.

Calcium phosphate nanoparticles (CPN) have long been regarded as potential drug and gene delivery vehicles due to their excellent biocompatibility, biodegradability, and colloidal stability, and they can encapsulate negatively charged therapeutic agents by chelating calcium ions while forming calcium phosphate nanocrystals^42,43^. CPN is the major component of bone and tooth enamel, and both Ca^2+^ and PO_4_^3−^ are found in the bloodstream at a relatively high concentration (1–5 mM)^44,45^. Lipid calcium phosphate (LCP) nanoparticles have been found to achieve both systemic delivery of drugs/genes to the lymphatic system and imaging of lymph node metastasis^46^. PEGylated calcium phosphate hybrid micelles enhance the in vivo accumulation of SiRNA in tumor tissues and promote their gene-silencing activity^47^. Calcium phosphate-based organic–inorganic nanocarriers are known for switching on photodynamic therapy in response to acidic environments^48^. Mn^2+^ within calcium phosphate nanoparticles of poly(ethylene glycol) shells has been found to act as an efficient magnetic resonance imaging (MRI) contrast agent that rapidly amplifies magnetic resonance signals in response to pH^49^.

**Fig. 2:**
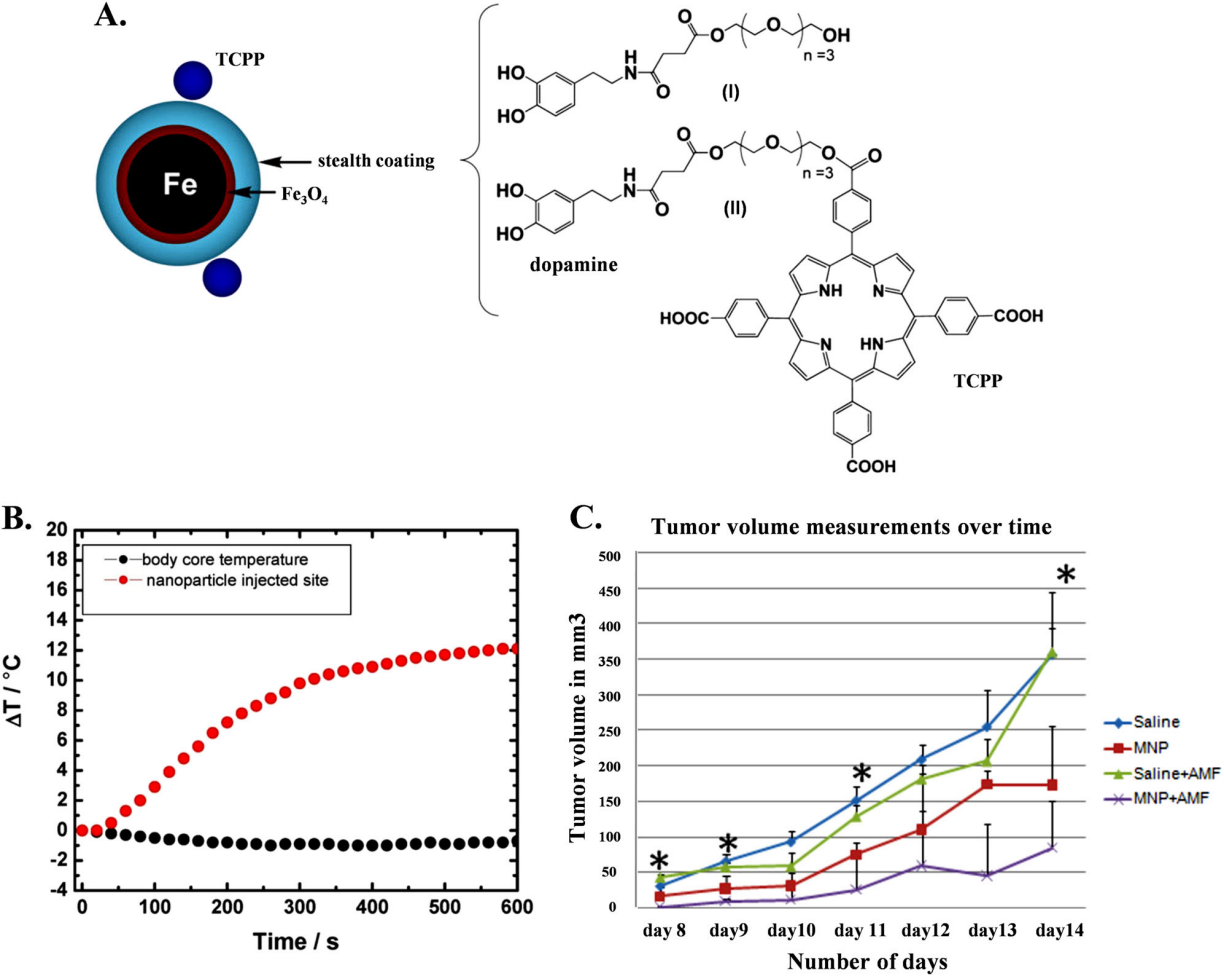
Effect of surface modification on magnetic nanoparticle on hypothermia to reduce tumor size. **a** Schematic presentation showing the composition of the 4-tetracarboxyphenyl porphyrin (TCPP)-labeled, dopamine-anchored tetraethylene glycol ligands coated bimagnetic Fe/Fe3O4 nanoparticles; **b** Graph illustrating the temperature profiles at the MNP injection site in the body core during alternating magnetic field (AMF) exposure, which is measured with a fiber optic temperature probe; **c** In vivo antitumor response after intratumoral injection of MNPs followed by AMF treatments. Graph demonstrates the relative changes in average tumor volumes over time of B16–F10 tumor bearing mice that were later injected with either saline or MNP intratumorally with or without AMF treatments^63^

Superparamagnetic iron oxide nanoparticles (SPIONs) are receiving increased attention for chemotherapy, hypothermia, magnetic resonance imaging (MRI), tissue engineering, cell and tissue targeting and transfection due to their intrinsic properties, such as inherent magnetism, visualization by magnetic resonance (MR) imaging, biocompatibility, guidance to target sites by means of an external magnetic field, heating to provide hyperthermia for cancer therapy and degradation into nontoxic iron ions in vivo. SPIONs are composed of an inner magnetic particle core (usually magnetite, Fe_3_O_4_, or maghemite, γ-Fe_2_O_3_) and a hydrophilic coating of polymers, such as polysaccharide, poly(ethylene glycol) (PEG), and poly(vinyl alcohol). Direct use of SPIONs without any polymer coating as an in vivo MRI contrast agent results in biofouling of the particles in blood plasma and particle agglomerations that are quickly sequestered by cells of the reticular endothelial system (RES), such as macrophages^50,51^. The coating helps to shield the magnetic particle from the surrounding environment and can also be functionalized by targeting ligands. These magnetic drug-bearing nanocarriers rely on external magnetic field guidance to reach their target tissue. Magnetic albumin microspheres (MM-ADR) in animal tumor models exhibit better responses than adriamycin alone both in terms of tumor size reduction and animal survival^52^. The enhanced efficacy of magnetic albumin microspheres in the targeted delivery of an anticancer agent compared to the pure drug in rat model is predominantly due to magnetic effects and is not due to the particle’s size or nonmagnetic holding^53^. SPION based MRI is a very powerful non-invasive tool in biomedical imaging, clinical diagnosis, and therapy. SPIONs potentially provide higher contrast enhancement in MRI and are much more bio-friendly than conventional paramagnetic Gd-based contrast agents^54,55^. Various methods of SPION preparation along with functionalization for targeted therapy and applications in cancer treatment have been reported^56^. Monocrystalline iron oxide nanoparticles (MION) and crosslinked iron oxide nanoparticles (CLIO) are two typical examples of dextran-coated SPIONs and have been widely used in vivo and in vitro MRI^57,58^. Anti-biofouling polymer coated thermally crosslinked superparamagnetic iron oxide nanoparticles (TCL-SPIONs) act as a novel MR contrast agent for in vivo cancer imaging^59^ and Cy 5.5-conjugated TCL-SPIONs act as a dual (MR/optical) cancer-imaging probe^60^. SPIONs have the potential to cure cancer by generating local heat when exposed to an alternating magnetic field. Cancer cells are susceptible to hyperthermia when the temperature increases to ∼43 °C for 30–60 min, which triggers apoptosis^61,62^. Porphyrin-tethered, dopamine-oligoethylene glycol ligand coated bimagnetic Fe/Fe_3_O_4_ nanoparticles act as a significant antitumor agent on murine B16-F10 mice with three short 10 min alternating magnetic field (AMF) exposures (Fig. 2)^63^. However, hyperthermia alone has not been found to be sufficient for cancer treatment, and it is often used as an adjuvant to other forms of therapy, such as surgery, radiotherapy, and chemotherapy^64^. Thus, recent research has focused on combining chemotherapy and hyperthermia using multifunctional SPIONs. Phospholipid-PEG coated SPIONs have the potential to concurrently deliver doxorubicin and generate heat for enhanced multimodal cancer treatment^65^.

#### Organic nanocarriers

Polymeric nanoparticles are solid, biocompatible, colloidal and often biodegradable systems with nanoscale dimensions. Polymeric nanoparticles are one of the simplest forms of soft materials for nanomedicine applications due to their facile synthesis and easy structural modification to allow desired properties to be built into the nanoparticle, such as surface modifications to improve drug loading efficacy, biodistribution, pharmacokinetic control and therapeutic efficacy^65,66^. Polymeric nanoparticles can be made from synthetic polymers, e.g., poly(lactic acid) (PLA), poly(ε-caprolactone) (PCL), poly(lactic-co-glycolic acid), *N*-(2-hydroxypropyl)-methacrylamide copolymer (HPMA) and poly(styrene-maleic anhydride) copolymer, or from natural polymers, such as gelatin, dextran, guar gum, chitosan, and collagen. Drugs can easily be encapsulated either through dispersion in the polymer matrix or conjugation/attachment to polymer molecules for their controlled delivery through surface or bulk erosion, diffusion through the polymer matrix, swelling followed by diffusion, or as a response to local stimuli. Synthetic polymers have the advantage of sustained release over a period of days to several weeks compared to the relatively shorter duration of drug release of natural polymers; their other benefits include the use of organic solvents and the requirement of typical conditions during encapsulation. Polymeric nanoparticles have therefore been widely investigated as drug delivery systems over the last few decades, including the clinical study of FDA-approved biodegradable polymeric nanoparticles, such as PLA and PLGA. A drug (doxorubicin) has been conjugated with dextran and subsequently encapsulated in a hydrogel using a reverse microemulsion technique reduce its cytotoxic effects and improve its therapeutic efficacy in the treatment of solid tumors^67^. Tamoxifen embedded PLGA nanoparticles, which were prepared using an emulsified nanoprecipitation technique, exhibit DNA cleavage potential and greater in vitro anticancer activity than the pure drug^68^. Multifunctional Taxol-loaded PLGA nanoparticles show chemotherapeutic and near-infrared photothermal destruction of cancer cells in vitro and in vivo^69^. However, by carefully manipulating the drug-to-polymer ratio, molecular weight, and nature of polymer, the extent and level of drug release from nanoparticles can be fine-tuned for effective cancer treatment.

Liposomes are small, spherical, self-closed structures with at least one concentric lipid bilayer and an encapsulated aqueous phase in the center. They have been widely used as drug delivery vehicles since their discovery in 1965 due to their biocompatible and biodegradable nature and their unique ability to encapsulate hydrophilic agents (hydrophilic drugs, DNA, RNA, etc.) in their inner aqueous core and hydrophobic drugs within the lamellae, which makes them versatile therapeutic carriers. Furthermore, amphiphilic drugs can also be loaded into the liposome inner aqueous core using remote loading methods, such as the ammonium sulfate method for doxorubicin^70^ or the pH gradient method for vincristine^71^. However, one of the major drawbacks of these conventional liposomes was their rapid clearance from the bloodstream. The development of stealth liposomes is underway by utilizing the surface coating of a hydrophilic polymer, usually a lipid derivative of polyethylene glycol (PEG), to extend the circulation half-life of liposomes from less than a few minutes (conventional liposomes) to several hours (stealth liposomes)^72^. Liposomes have the potential to target specific cells through both active and passive targeting strategies. PEGylated liposomes have been found to be more effective at passively targeting cancer cells both in vitro and in vivo than conventional liposomes, and moreover, PEGylated liposomes exhibit a high degree of nuclear transfection. Liposomal antisense oligonucleotides (ASO) have been found to be effective for the inhibition of pump and nonpump resistance of multidrug resistant tumors^73^. Ligand-targeted liposomes have been found to promote the internalization of liposome-drug conjugates into specific target cells both in vitro and in vivo, and the vectors can be designed to release their contents in the enzyme rich, low pH environment of endosomes and lysosomes using pH-triggered approaches^74,75^. Targeted zwitterionic oligopeptide liposomes exhibit enhanced tumor cell uptake, improved cytoplasmic distribution and enhanced mitochondrial targeting^76^. A couple of clinically approved liposomal products are on the market and more are under clinical development (Table 1). Interestingly, all of these approved liposomal nanocarriers act through passive targeting strategies, whereas few targeted formulations have progressed into the clinic. Ceramide liposomes show an inhibitory effect on peritoneal metastasis in a murine xenograft model of human ovarian cancer and suppress the cell mortality of prometastatic factor, which is promoted by epithelial growth factor^77^. Vascular endothelial growth factors (VEGF), which are often over expressed in many cancers associated with angiogenesis, are induced at a transcriptional level and suppress VEGF and other genes using RNA interference (RNAi), which is considered to be a novel therapeutic strategy in the silencing of disease-causing genes. Chitosan-coated liposomal formulations, such as the siRNA delivery system, is effective at achieving gene silencing efficiency^78^. Paclitaxel containing A7RC peptide modified liposomes acts as an antimitotic chemotherapeutic drug, which can be a promising strategy for promoting antitumor and antiangiogenic therapies^79^.

##### Protein-based nanocarriers

Albumin is a protein that can be obtained from a variety of sources, including egg white (ovalbumin), bovine serum (bovine serum albumin, BSA), and human serum (human serum albumin, HSA), and is available in soybeans, milk, and grains. Albumin-based nanocarriers have several advantages, such as easy preparation, a high binding capacity for various drugs, nontoxic, non-immunogenic, biocompatible, and biodegradable properties, and along half-life in circulating plasma. The presence of functional groups (amino and carboxylic groups) on albumin nanoparticles surfaces makes it easy to bind targeting ligands and other surface modifications^80^. Doxorubicin loaded human serum albumin (HSA) nanoparticles have been found to have better in vitro antitumor efficacy than the pure drug against neuroblastoma cell lines (UKF-NB3 and IMR 32)^81^. Paclitaxel-loaded bovine serum albumin (BSA) nanoparticles, which are prepared using a desolvation technique, followed by folic acid decoration, have been found to target human prostate cancer cell line (PC3) effectively^82^. Albumin is a natural carrier of hydrophobic molecules (hormones, vitamins and other plasma constituents) through favorable, noncovalent reversible binding and helps with their transportation in body fluids and release at the cell surface. Moreover, albumin can bind to the glycoprotein (gp60) receptor and mediate the transcytosis of albumin-bound molecules^83,84^. Abraxane (nab-paclitaxel; paclitaxel-albumin nanoparticle), which has an approximate diameter of 130 nm, is the first FDA-approved commercial product based on the nab platform that has shown significant benefits in the treatment of metastatic breast cancer. Several other nab-technology based chemotherapeutics, such as nab-docetaxel, have already entered into clinical trials.

##### Micelles as a drug carrier

Micelles are spherical or globular colloidal nanoscale systems formed by self-assembly of amphiphilic block copolymers in an aqueous solution, resulting in a hydrophobic core and a hydrophilic shell. They belong to a group of amphiphilic colloids that can be formed spontaneously under certain concentrations (critical micelle concentration; CMC) and temperatures. The hydrophobic core serves as a reservoir for hydrophobic drugs, whereas the hydrophilic shell stabilizes the hydrophobic core and renders both polymer and hydrophobic drugs water soluble, making the particle an appropriate candidate for i.v. administration. The drugs are incorporated into a polymeric micelle through physical, chemical, or electrostatic interactions^65^. The first polymeric micelle formulation of paclitaxel, Genexol-PM (PEG-poly(d,l-lactide)-paclitaxel), is Cremophor-free polymeric micelle-formulated paclitaxel, which can be administered without reactions and shows a favorable toxicity profile with advanced refractory malignancies^85^. Multifunctional star-shaped polymeric micelles, based on four-arm disulfide linked poly(ε-caprolactone)-poly(ethylene glycol) amphiphilic copolymers coupled with folate ligands,exhibit high stability and sustained release, whereas prompt release can occur in an acidic environment^86^. Doxorubicin is encapsulated into cationic 1,2-dioleoyl-3-trimethylammonium propane/methoxy poly(ethyleneglycol) (DPP) nanoparticles to form micelles for intravesical drug delivery and has shown an anticancer effect against bladder cancer^87^. Cholesterol-modified mPEG–PLA micelles (mPEG–PLA-Ch) exhibit high encapsulation efficiency and reduce tumor size considerably compared to the pure drug (curcumin)^88^. Phenylboronic acid (PBA) can selectively recognize sialic acid (SA), showing a high affinity for targeting sialylated epitopes that are over expressed on cancer cells, and oxaliplatin incorporated micelles exhibit enhanced tumor-targeting ability through specific interactions with SA (as confirmed using fluorescence spectroscopy), leading to an attractive strategy for increasing the efficiency of chemotherapies^89^. Gilbreth *et al.*^90^ explored the use of lipid- and polyion complex-based micelles for the rapid generation of multivalent agonists targeting tumor necrosis factor receptors, and the micelles showed promising therapeutic efficacy.

##### Self-assembly as a drug carrier

**Fig. 3:**
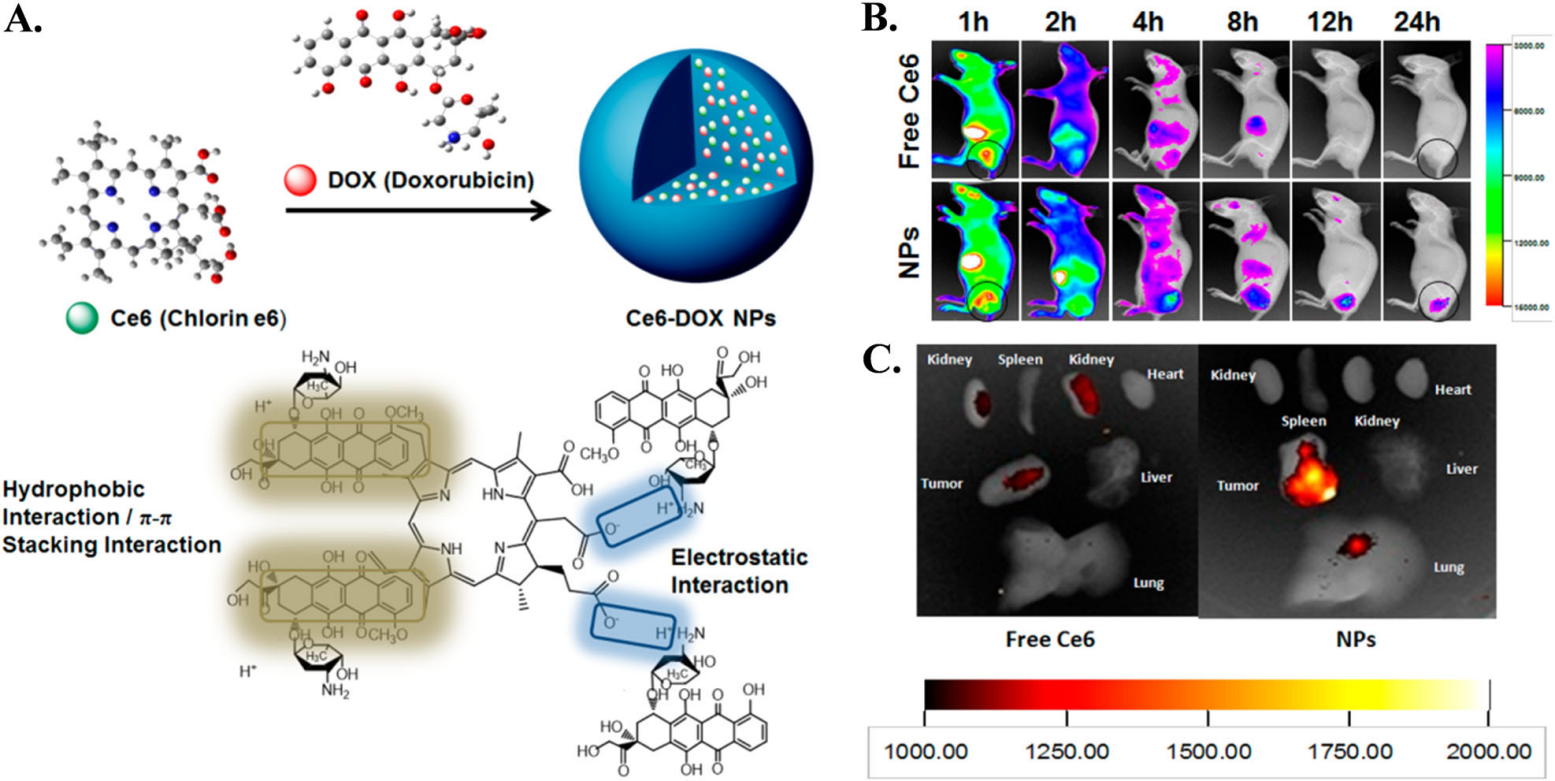
Co-asembly of drug and photo photosensitizer for better imaging of tumor size during treatment. **a** Schematic representation of carrier-free nanoparticles (NPs) via co-assembly between DOX and Ce6; **b** In vivo fluorescence images of free Ce6 solution and Dox/Ce6 nanoparticles (NPs). The areas in the black circles represent tumor tissue; **c** Representative ex vivo fluorescence imaging of tumor and organs excised from Balb/c nude mice xenografted MCF-7 tumor at 24 h post-injection^92^

Molecular self-assembly is a free energy driven process that spontaneously organizes molecules into ordered structures in multiple geometries. Therefore, self-assembly is a very attractive approach to constructing nanoscale based bioactive materials due to its straightforward use in biomedical applications, including tissue engineering, regenerative medicine and drug delivery. The great advantage of self-assemblies in its structural features, which can be tuned through molecular chemistry and environmental conditions (pH, ionic strength, solvents, and temperature)^91^. Self-assembly of the photosensitizer (chlorine e6, Ce6) and a chemotherapeutic agent (doxorubicin) linked with electrostatic, *π*−*π* stacking and hydrophobic interactions are designed to inhibit tumor recurrence (Fig. 3a). Intravenously administeredfree Ce6 and NPs were distributed throughout the body, whereas the administered self-assembly drugs accumulated in the tumor site exclusively (Fig. 3b). Ex vivo imaging of excised tumors further confirmed higher drug accumulation in tumors with NPs than with free Ce6 solution (Fig. 3c)^92^.

The switchable aptamer-diacyllipid conjugates, formed by the self-assembly of an aptamer switch probe-diacyllipid chimera, showed good results in molecular imaging for bioanalysis, disease diagnosis and drug delivery^93^. Upon target binding, the conformation of switchable aptamer was altered, resulting in the restoration of a fluorescence signal. The cellular transport of functional d-peptide derivatives relies on the use of taurine triggered intracellular self-assembly of the d-peptide derivative. Intracellular nanofibers formed by enzyme-instructed self-assembly can disrupt actin filaments and enhance the activity of cisplatin against drug-resistant ovarian cancer cells by controlling the fate of live cells^94^. Tumor-targeted delivery of siRNA by self-assembled nanoparticles is obtained by mixing carrier DNA, siRNA, protamine and lipids, followed by post-modification with poly(ethylene glycol) and a ligand, anisamide. Forty percent tumor growth inhibition was achieved by treatment with targeted nanoparticles, and complete inhibition lasted for 1 week when combined with cisplatin^95^.

##### Supramolecules as a delivery vehicle

Supramolecules are an assembly of two or more molecular entities stabilized by weak and reversible noncovalent interactions, such as hydrogen bonding, metal coordination, hydrophobic attractions, van der Waals forces, *π*−*π* and electrostatic interactions. Therefore, they are expected to function as a carrier in drug delivery designs. Supramolecular systems can provide vehicles for the encapsulation and targeted delivery of therapeutic agents or bioactive materials^96^. The toxicity of traditional anticancer drugs, such as doxorubicin, can be repressed using amphiphilic dendrimers that generate supramolecular micelles for cancer therapy^97^. DOX-loaded supramolecular polymersomes exhibit prolonged circulation in the bloodstream, and in vivo studies show that they have better antitumor efficacy towards cancerous HeLa cells with relatively lower cytotoxicity^98^. Cyclodextrin containing cationic polymer-based supramolecular hydrogels show reduced cytotoxicity compared to their non-CD-containing polymer counterparts^99^. The temperature responsive behavior of poly(*N*-isopropylacrylamide) supramolecular micelles and rapid drug release rate are due to induced physical crosslinking; however, these supramolecular micelles demonstrate excellent biocompatibility against most cell lines^100^. Dankers*et al.*^101^ introduced a new concept of transient supramolecular networks in which macroscopic rheological and materials properties are tuned by controlled microscopic supramolecular interactions; these interactions are responsible for network formation and lead to promising protein delivery carriers in regenerative medical applications, such as the proof-of-concept showed in kidney regeneration. Real-time observation of drug distributions by supramolecular nanocarriers for the treatment of pancreatic tumors has been investigated to obtain feedback on therapeutic efficacy at an early stage. There is no drug accumulation in healthy pancreas, which is supported by the strong diagnostic and anticancer effect of supramolecular micelles^102^. Tumor-targeted drug delivery systems based on supramolecular interactions between iron oxide–carbon nanotubes and polyamidoamine–polyethylene glycol–polyamidoamine (PAMAM–PEG–PAMAM) linear-dendritic copolymers are used as promising systems for future cancer therapy with low drug doses^103^. Porphysome nanovesicles are formed through the self-assembly of porphyrin-lipid bilayers that generate unique fluorescence, photothermal and photoacoustic properties. The drug loading capacities of porphysomes to destroy tumors by releasing heat are due to their ability to absorb light in the near-infrared region, which generates a photoacoustic signal that can then be converted into an image. Porphysomes are stable for months when stored in aqueous solutions, but they are prone to enzymatic degradation when incubated with detergent and lipase. The mice in the porphysome and laser-treated group developed eschars on the tumors (which healed) and their tumors were destroyed^104^. Muhanna*et al.*^105^ demonstrated the effectiveness of porphysome nanoparticles to enhance fluorescence and photoacoustic imaging of oral cavity carcinomas in rabbit and hamster models along with tumor-localized photothermal therapy (PTT). PTT can be precisely guided by both fluorescence imaging (control of laser placement and tumor delineation) and photoacoustic imaging (tumor margin delineation and assessment of effective PTT doses) for effective therapeutic efficacy. A tailor-made porphyrin based micelle, self-assembled from a hybrid amphiphilic polymer comprising polyethylene glycol, poly (d,l-lactide-co-glycolide) and porphyrin, loaded with two chemotherapeutic drugs with synergistic cytotoxicity showed a tendency to accumulate in tumor cells. Drug-loaded micelles improved therapeutic efficacy against triple-negative breast cancer through the synergistic effects of photothermal therapy, DOX, and TAX with biocompatible polymers and porphyrin^106^.

### Hydrogel as a delivery vehicle

**Fig. 4:**
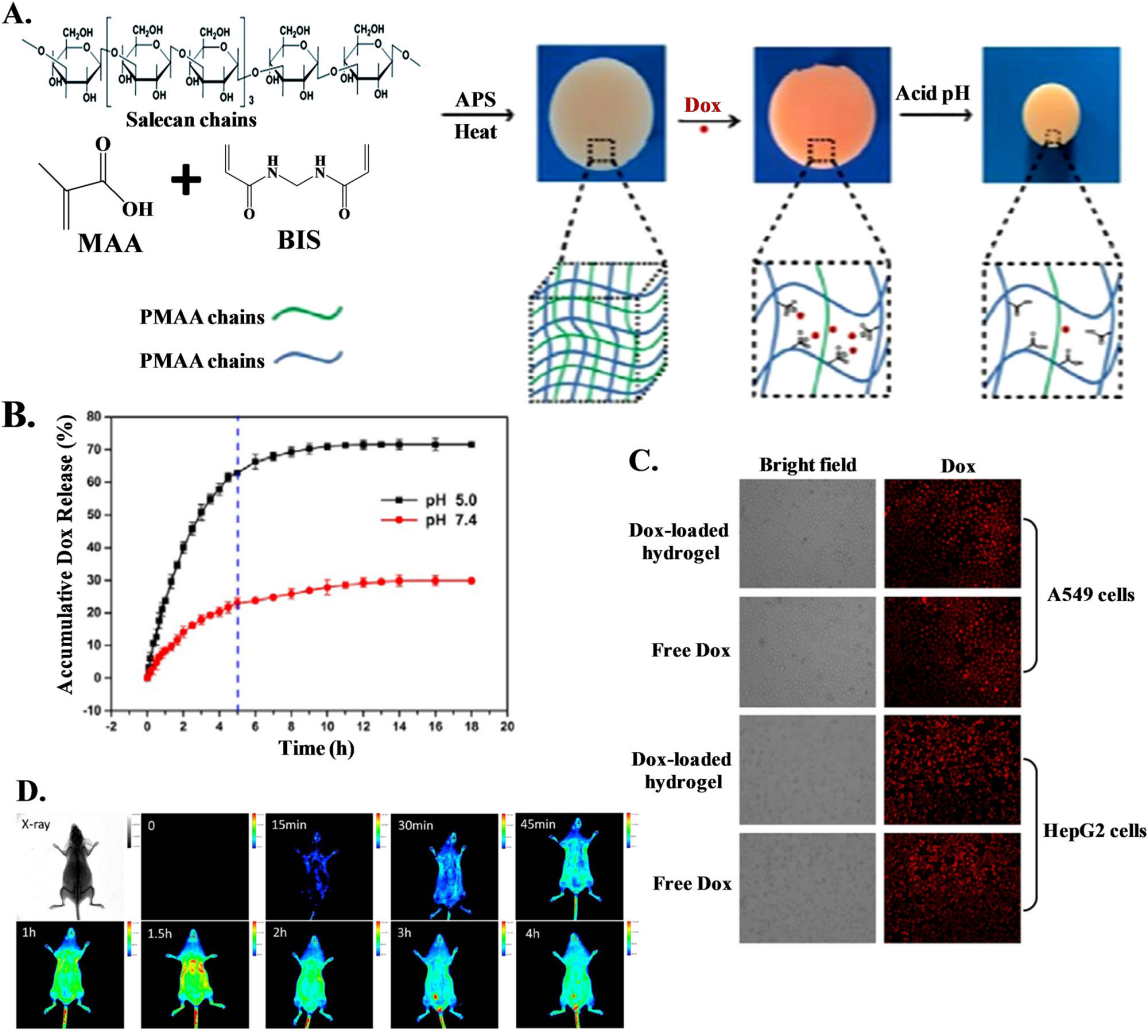
Control delivery of drug using hydrogel as vehicle. **a** Illustration of the preparation and drug release of Salecan/PMAA semi-IPN hydrogels; **b** In vitro Dox release behaviors from the semi-IPN sample at two different pH values of 5.0 and 7.4; **c** Fluorescent microscopy images of A549 and HepG2 cells after 4 h of incubation with 6 μg/mL free Dox solutions and the extract liquid of Dox-loaded hydrogel;^112^ **d** Intravital real-time fluorescence images of ICR mice injected with FITC-labeled PMAA nanohydrogels^126^

Hydrogels are three-dimensional (3D) polymeric and hydrophilic networks that can absorb large amounts of water or biological fluids. Hydrogels are thermodynamically compatible with water, which allows them to swell in aqueous media^107^. Hydrogels are widely used for numerous applications in the pharmaceutical and medical fields, e.g., as biosensors, materials for contact lenses, artificial skin, and lining for artificial hearts. Moreover, they can also be used for 3D cell culture and as drug delivery vehicles^108,109^. Hydrogels are efficient candidates for controlled release, bioadhesive and/or targeted drug delivery as they can encapsulate biomacromolecules, including proteins and DNA, and hydrophilic or hydrophobic drugs^110^. Hydrogel-based drug delivery systems can be used in different ways for oral, rectal, ocular, epidermal, and subcutaneous applications^107^. The key success of hydrogel development is in situ gelation. Hydrogels can be prepared by introducing non-reversible covalent bonds via self-assembly either through reversible interactions or non-reversible chemical reactions or by UV/photo polymerization. The gelation process is time and concentration dependent and can be triggered by an external stimulus, such as pH, temperature, or light^111^. Hydrogels have been found to be biocompatible with negligible cytotoxicity and can be utilized as a delivery platform when accessed with the normal cell line COS7 and cancer cell lines HepG2 and A549. A variety of innovative semi-interpenetrating polymer network (semi-IPN) hydrogels consisting of salecan and poly(methacrylic acid) (PMAA) are formed via free-radical polymerization for controlled drug delivery (Fig. 4a)^112^. Drug release is facilitated under acidic conditions as protonated PMAA disrupts the electrostatic interaction between DOX and the hydrogel (pH < 5.5), favoring drug release compared the conditions at to pH ~ 7.0 (Fig. 4b). Due to this factor, pH ~ 5 is considered representative of simulated cancer environments. Successive exposure to a different release media at pH 7.4 and 5.0 causes pH-dependent ‘off-on’ switching of drug release. Cellular uptake of DOX released from drug-loaded hydrogels has effectively been shown in A549 and HepG2 cells, showing great promise for hydrogels to be utilized as a vehicle for anticancer drug delivery (Fig. 4c). Poly(vinyl pyrrolidone) stabilized fluorescent red copper nanoclusters can be converted into hydrogel nanocarriers through crosslinking with poly(vinyl alcohol) to deliver the anticancer drug cisplatin (CP) to cervical cancer cells (HeLa), thereby inducing apoptotic cell death^113^. The high encapsulating efficiency is attributed to molecule loading on the surface and inside the hydrogel particle, followed by strong interactions using various functionalities, such as −COOH. The slow release of CP at physiological pH is due to stronger bonding between the drug molecule and the hydrogel, which can be disrupted at acidic pH, favoring fast release. The significant decrease in cell viability in the presence of drug-loaded carriers as opposed to free drug molecules reveals the combination of Cu NC−hydrogel composites and CP as a potential material for the design of new chemotherapeutic agents. Doxorubicin loaded poly(methacrylic acid) (PMAA) hydrogel cubes and spheres are capable of both intracellular degradation and pH-responsiveness by introducing cystamine crosslinks within networks^114^. The membrane adhesion process in the initial step of cell internalization is greatly affected by the shape of the particles, and hydrogel spheres exhibit 12% higher cell cytotoxicity than cubes using HeLa cells for 10 h. Shape and pH-sensitive ‘intelligent’ 3D networks with programmable shape-regulating behaviors are promising candidates for the controlled delivery of chemotherapeutics. Doxorubicin encapsulated poly(vinylcaprolactam) (PVCL)-based biodegradable microgels have been designed for stimuli-triggered drug release in acidic or reducing environments^115^. DOX-loaded microgels exhibit efficient antitumor activity to HeLa cells against nontoxic blank microgels. Supramolecular hybrid hydrogels of α-cyclodextrin and polyethylene modified gold nanocrystals exhibit pH-dependent sustained release of DOX through host–guest interaction^116^. Tetrapeptide-based thermoreversible, pH-sensitive hydrogels have been prepared for the slow release of anticancer drugs at physiological pH^117^. Hexamethylene diisocyanate (HDI) reacts with Pluronic F127 as a chain extender to form a copolymer, and subsequent incorporation of hyaluronic acid (HA) has been used to develop a composite hydrogel system with a sol–gel transition at 37 °C, leading to the formation of a nanocomposite injectable hydrogel for drug delivery with controlled release^118^.

### Hybrid materials for controlled drug delivery

Nanoscale dimension hydrogel particles are often called ‘nanogels’ and are formulated either by physically or chemically crosslinked hydrophilic polymers^10,119^. Nanogels have been recently exploited in various fields, including diagnostics, chemical and biochemical sensing, tissue engineering, and cancer imaging, especially as drug delivery vehicle^120–123^. Nanogels offer several advantages in therapeutic delivery in comparison to existing nanocarriers: (i) high drug loading capacity; (ii) higher storage stability than liposomes and micelles; (iii) controlled and sustained drug release; (iv) ease of synthesis; (v) response to external stimuli; and (vi) low inherent toxicity^124,125^. Nanogels act like a soft material when exposed to aqueous media with high water content. Protease/redox/pH stimuli-responsive PEGylated poly(methacrylic acid) PMAA nanohydrogels have been synthesized using cystamine as crosslinker for targeted anticancer drug delivery^126^. The cumulative release profile indicates greater release in acidic media (pH ~ 5.0) and reducing environments (GSH). Intravital real-time fluorescence image analysis indicates the quick accumulation and maintenance of FITC-labeled PMAA nanohydrogels in the kidney, liver, and other organs, such as the heart, lung, and spleen; after 30 min of administration, extended in vivo blood circulation lifetimes have been shown using PEGylated FITC-labeled PMAA nanohydrogels (Fig. 4d). Yang *et al.*^127^ prepared poly(*N*-isopropylacrylamide)-ss-acrylic acid (P(NIPAM-ss-AA)) nanogels based on NIPAM and AA crosslinked by *N*,*N*ʹ-bis(acryloyl) cystamine (BAC) through precipitation polymerization, which exhibited pH/redox dual-responsive DOX release in vitro and in tumor cells. Animal studies have demonstrated the efficient penetration of DOX-loaded nanogels with fewer side effects, indicating a prospective platform for intracellular controlled drug release in cancer therapy. Bovine serum albumin (BSA) and chitosan (CS) nanogels prepared via a green self-assembly technique exhibit slow release with lower cytotoxicity^128,129^. Biocompatible and pH-responsive self-assembled nanogels of chitosan-graft-poly(*N*-isopropylacrylamide) have been used as a model tumor-targeting delivery system and had greater activity in acidic media^130^. Alginate-PAMAM dendrimer-based hybrid nanogels have been developed for drug delivery to cancer cells, and they showed pH-dependent release behavior in a sustained way^131^. Nanocarriers have been found to release more drug in acidic environments (mimicking tumor microenvironments) than in physiological conditions.

Nanotechnology is a rapidly growing research area in the field of catalysts, biosensors, bioimaging, energy devices, and targeted drug delivery^132–134^. The large surface-to-volume ratio of nanoparticles and their size, ability to carry other compounds, binding ability and their adsorption properties make them suitable for biomedical applications. Nanoparticles can also improve bioavailability, protect drugs from degradation and control release rates, i.e., provide sustained drug release. These unique characteristics of nanoparticles offer a viable platform for their use as an effective drug delivery system^135^. Biodegradable carboxymethyl cellulose/graphene oxide (CMC/GO) nanohybrid hydrogel beads physically crosslink with FeCl_3_.6H_2_O have been used for the controlled release of an anticancer drug (DOX)^136^. The *π*–*π* stacking interaction between GO and DOX caused higher drug loading efficiency. The release profile from hydrogels was highly pH-dependent, based on hydrogen bonded interactions and exhibited a faster release at pH ~ 6.8 than in slightly basic media (pH ~ 7.4). Furthermore, greater amounts of filler/GO reduced the release rate because of enhanced interactions between the components. Halloysite nanotubes embedded hybrid hydrogels of poly(hydroxyethyl methacrylate) with sodium hyaluronate were very effective for colon cancer drug delivery^137^. Anticancer drugs, such as 5-fluorouracil (5-FU), have been encapsulated not only in hydrogel networks but also in halloysite nanotubes using an equilibrium swelling method, followed by pulling and breaking the vacuum. In vitro release of 5-FU from nanohybrid hydrogels exhibited pH-dependent controlled release following diffusion-controlled non-Fickian transport behavior. 5-FU was also intercalated within the gallery of natural montmorillonite (Mt) clay, which could be compounded using alginate (Alg), followed by a coating with chitosan (CS), to prepare a complex drug release system with controlled release behavior^138^. The release rate of 5-FU was found to be retarded when using an Alg–CS/5-FU/Mt nanohybrid system in gastric and intestinal environments. Hybrids of nanoclay and chitosan–polylactide blends also released paclitaxel in a pronounced manner in basic conditions compared their release in acidic environments^139^. Biodegradable poly(ε-caprolactone)nanohybrid scaffolds with organically modified nanoclay, which were prepared through an electrospinning technique, exhibited sustained delivery of an anticancer drug (dexamethasone) vis-à-vis pure polymer by creating a maze or ‘tortuous path’ that retarded the diffusion of the drug from the matrix in the presence of a two-dimensional filler^140^. Biocompatible polyurethane nanohybrids using an aliphatic diisocyanate and aliphatic chain extender with varying chain lengths and 2D nanoclay was designed for sustained drug delivery of an anticancer drug in which the tortuous path was created through larger crystallites from self-assembly of a hard segmented zone^141^. Graphene-based polyurethane nanohybrids have been prepared by grafting long chain polyurethane onto the surface of functionalized graphene oxide for sustained drug delivery of an anticancer drug (dexamethasone)^142^. Chemically tagged amine- and sulfonate-functionalized graphene within long chain polyurethane molecules has been developed for the sustained release of dexamethasone^143,144^. A hard segment in pure polyurethane was responsible for delayed drug release, whereas the self-assembled structure and graphene moieties acted as a barrier for the diffusion of loaded drugs in nanohybrids. Several other polymer nanoparticle hybrid systems have been reported as sustained release systems for cancer therapy using different drugs, such as DOX, 5-FU, and MTX^145–149^. The dual-administration of DOX with MTX had higher cytotoxicity towards T47D breast cancer cells than free dual drug forms. Dual anticancer drug-loaded antibacterial smart polymer nanohybrids have the potential to be used for combination cancer therapy.

## Targeted delivery: mechanistic pathway

**Fig. 5:**
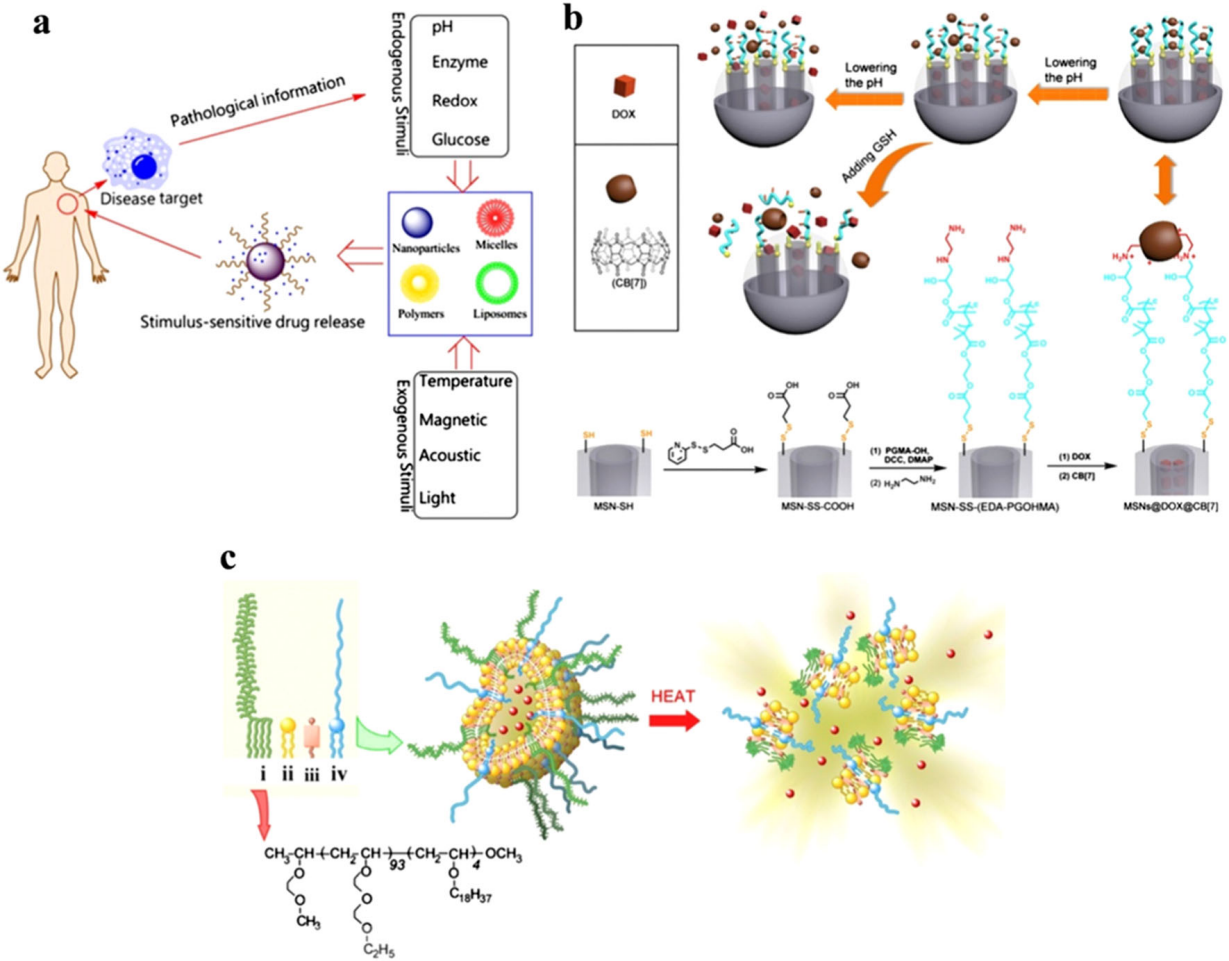
Stimuli-responsive targeted delivery of therapeutic agents. **a** Schematic illustration of stimuli-responsive DDS;^148^ **b** Schematic diagram of pH and GSH dual-responsive dynamic crosslinked supramolecular network on MSN-SS-(EDA-PGOHMA) and synthetic route with CB^7^ assembly;^151^ **c** Design of temperature-sensitive liposomes composed of thermosensitive poly(EOEOVE)-OD4 (i), membrane-forming EYPC (ii), membrane-stabilizing cholesterol (iii), and highly hydrophilic and nontoxic PEG-lipid (iv). Heat-triggered release of DOX from liposomes is illustrated with the structure of poly(EOEOVE)-OD4^153^

Target-specific deliveries of therapeutic agents are based on stimuli-responsive factors induced by either endogenous (pH, redox, enzyme) or exogenous stimuli (temperature, acoustic, light) as shown in Fig. 5a^150^. pH-sensitive chitosan-based supramolecular gel is used for oral drug delivery of insulin. The pH sensitivity of the nanogel protects insulin while it is in the stomach, and the bioadhesivity of chitosan enables prolonged contact with the intestinal mucosae to increase the absorption of insulin^151^. The drug delivery platform based on mechanized silica nanoparticles (MSNPs), which consists of MSNs vehicles, acid-cleavage intermediate linkages and reversible supramolecular nanovalves, was devised to achieve multimodal controlled release of two drugs, gemcitabine (GEM) and doxorubicin (DOX), by arranging the order of stimuli in sequence. The release time and dosage of GEM are precisely controlled via external voltage, whereas subsequent acid treatment triggers the release of DOX, which is attributed to breakage of the intermediate linkages containing ketal groups^152^. Dynamic crosslinked supramolecular networks of poly(glycidyl methacrylate) derivative chains on mesoporous silica nanoparticles respond well against the dual stimuli of pH and glutathione-(GSH-) linkage, which control the release of anticancer drug doxorubicin hydrochloride (DOX) under a simulated tumor intracellular environment (pH = 5.0, CGSH = 2~10 mM). Disassembly of the crosslinked polymer network occurs by lowering the pH, and cleavage of disulfide bonds efficiently promotes drug release kinetics (Fig. 5b)^153^. Glutathione disulfide (GSSG) is the oxidized form of glutathione (GSH), which is the major endogenous antioxidant. Glutathione protects biological systems from oxidizing factors, such as reactive oxygen species, by terminating them; GSH is oxidized to GSSG, and then it is reduced back to GSH by glutathione reductase (GR). The unique antimetastatic mechanism of glutathione disulfide (GSSG) based liposomes completely prevents cell detachment and migration and significantly inhibits cancer cell invasion and has been confirmed as a potential treatment for cancer metastasis^154^. Temperature-sensitive liposomes with temperature-sensitive amphiphilic polymer poly(EOEOVE)-OD4 are used for tumor-specific chemotherapy. DOX encapsulated liposomes are administered intravenously into tumor bearing mice, and tumor growth is significantly suppressed only when the tumor site is heated to 45 °C for 10 min after 6–12 h of injection (Fig. 5c)^155^. Biocompatible poly(N-(2-hydroxypropyl-methacrylamide) (PHPMA) functionalized cyclodextrin (CD) is the building block that houses two guests, *e.g*., poly(N,N-dimethylacrylamide) (PDMAAm) and poly(N,N-diethylacrylamide) (PDEAAm), prepared via reversible addition−fragmentation chain transfer (RAFT) polymerization and can form a well-defined supramolecular ABA triblock copolymer responsive to UV light and temperature. CD-based host/guest complexes show thermoresponsivity due to the negative enthalpy of complex formation. The application of these stimuli leads to the disassembly of the triblock copolymer, which has been shown to be reversible, and is ultimately responsible for regulated delivery. In case of PDEAAm, temperature-induced aggregation is observed after heating above the cloud point of the PDEAAm block^156^. Tripeptide Lys–Phe–Gly (KFG), a biologically important tripeptide, is spontaneously self-assembled into well-defined nanostructures in aqueous media, showing an exciting phenomenon of reversible and concentration dependent switching of nanostructures between nanovesicles and nanotubes as evidenced by dynamic light scattering, transmission electron microscopy and atomic force microscopy studies. The tripeptide vesicles have inner aqueous compartments and are stable at pH 7.4, but they rupture rapidly at pH 6. The pH-sensitive response of the vesicles is exploited for delivery of a chemotherapeutic anticancer drug (doxorubicin), resulting in enhanced cytotoxicity for both drug-sensitive and drug-resistant cells. The absence of the KFG sequence in the receptor polypeptide chain of tyrosine kinase nerve growth factor (Trk NGF) strongly affects the activation of signaling cascades. Efficient intracellular release of the drug is confirmed by fluorescence-activated cell sorting analysis, fluorescence microscopy, and confocal microscopy^157^. A combination of an aptamer for target recognition and enzyme phosphatidylcholine 2-acetylhydrolase (PLA2) for rupture of lipid bilayer of liposomes containing uranin and gadopentetic acid (GdDTPA) as signaling agents have been investigated for fluorescence and MRI detection. Thus, aptamer−PLA2 triggers the release mechanism via the target-responsive liposome system for signal transduction and selective recognition of biological molecules^158^.

### Magnetic field for cancer treatment

**Fig. 6:**
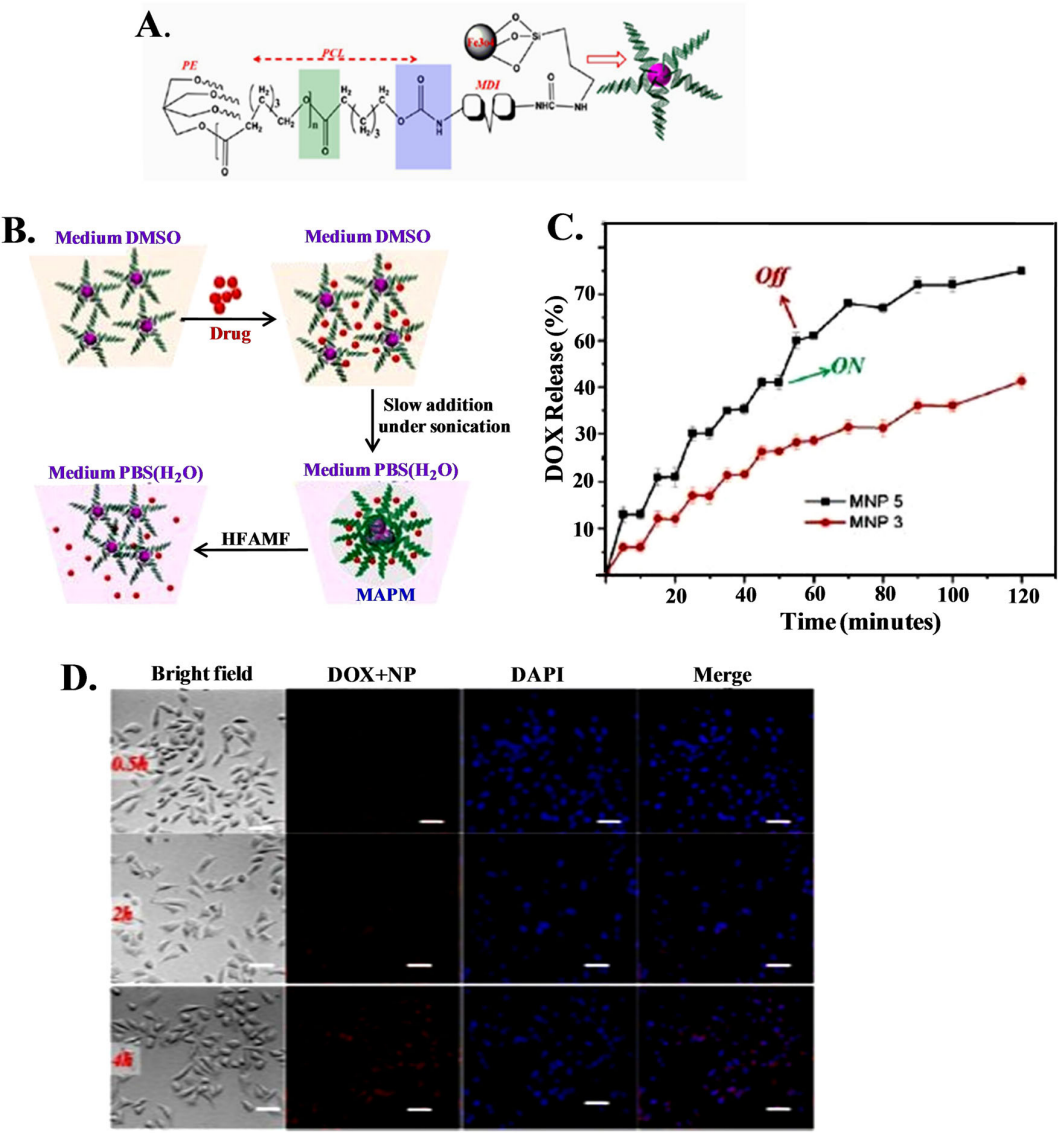
Reslease and cellular uptake of drug using magnetic nanoparticles under magnetic field. **a** Schematic representation of a four armed PE−PCL immobilized magnetic nanoparticle (MNP); **b** Schematic representation of DOX-loaded MNP and DOX release under the influence of high field alternating magnetic field (HFAMF); **c** The release kinetics of MNP 3 (particle size of 3 nm) and MNP 5 (particle size of 5 nm) under the influence of HFAMF at 37 °C; **d** Cellular uptake study of DOX-loaded MAPM on HeLa cell in the presence of a static magnetic field where the nucleus was stained by DAPI (blue)^157^. The scale bar is 40 μm

Magnetic (micro- or nanoparticles) materials were explored a couple of decades ago as potential carriers for specific drug targeting. External magnetic fieldscan be used as a responsive drug delivery system to transport drugs to tumor sites. Recently, superparamagnetic Fe_3_O_4_ magnetic nanoparticles have been synthesized through grafting using four armed pentaerythretol poly(ε-caprolactone) in the form of micelles for magnetically targeted controlled drug (DOX) delivery (Fig. 6a)^159^. The loading of DOX into the nanoparticle and its release under the influence of high frequency alternating magnetic fields is schematically shown in Fig. 6b. The release profiles at the two different temperatures are not remarkable, whereas drug release is considerable (51.5%) under the influence of a magnetic field for 1 h (Fig. 6c) as the self-assembled structure ruptures under a strong magnetic field. This form of magnetically controlled DOX release is quite advanced in magnetically active polymeric micelles and is superior from a patient compliance viewpoint compared to other conventional methods used for drug delivery (diffusion, pH, thermal response, etc.). The efficacy of magnetic fields for drug release is indicated by effective intercellular uptake after only 0.5 h of incubation in the presence of a magnetic field with no incorporation of the drug in absence of the field (Fig. 6d). A magnetically driven paclitaxel delivery system has been designed by incorporating iron oxide and a drug in a palmitoyl chitosan matrix through a nanoprecipitation method for controlled drug delivery under magnetic field^160^. Enhanced cell (MCF-7) death occurs due to the hyperthermic effects of magnetic nanoparticles in the presence of an external magnetic field, resulting in a biocompatible and biodegradable carrier for the precise delivery of powerful cytotoxic anticancer agents. A dramatic change in the amount of drug release is found when the remote magnetic field is switched ‘on’ and ‘off’ using silica magnetic nanocapsules containing camptothecin (hydrophobic) and doxorubicin (hydrophilic) in drug enriched areas near mouse breast tumors, and the nanocapsules are effective at reducing tumor cell growth^161^. Magnetic carriers for drug delivery using superparamagnetic nickel ferrite nanoparticles functionalized with poly(vinyl alcohol), poly(ethylene oxide) and poly(methacrylic acid) (PMAA) and subsequently conjugated with doxorubicin anticancer drug have significantly enhanced the release rate under magnetic fields by creating mechanical deformation, which generates compressive and tensile stresses to eject drug molecules^162^.

### Electric field for cancer therapy

**Fig. 7:**
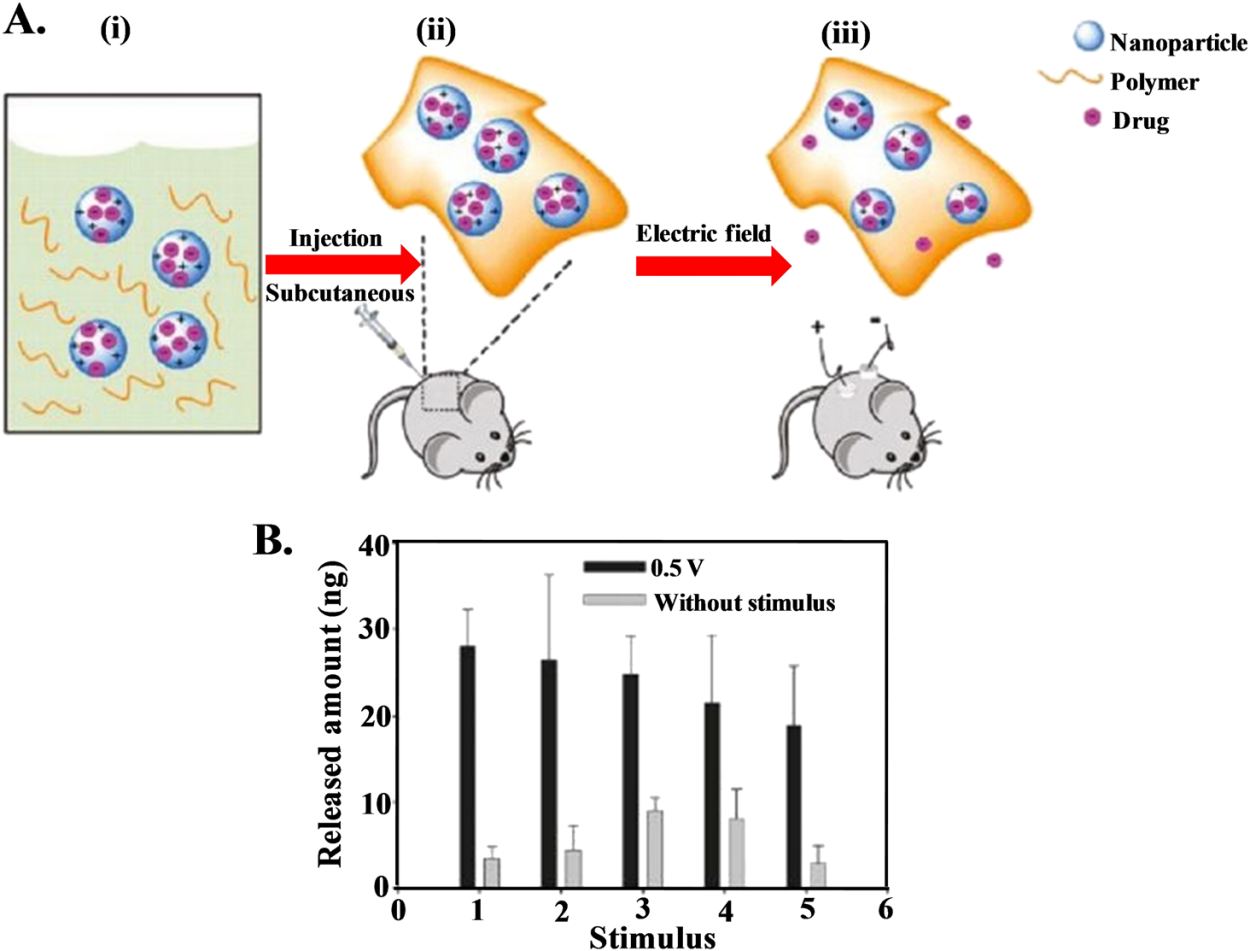
Electric field guided control release of drug. **a** General scheme for the application of this system: (i) the nanoparticle-polymer solution is (ii) subcutaneously injected into a mouse, followed by (iii) application of a DC electric field to induce the release of drug cargo inside the nanoparticles; **b** Released amount of daunorubicin in PBS (pH 7.2) following an applied voltage (0.5 V) duration of 10 s, repeated every 5 min^176^

Attention is being given to stimuli-responsive or ‘smart’ biomaterials in the fields of biotechnology and biomedicine^163–167^. Stimuli-responsive materials, which respond to heat^168,169^, pH^170,171^, light^172,173^, enzymes^174,175^, and magnetic fields^176,177^, are widely used in the biomedical arena. Electrical signals are easier to generate and control than other stimuli. Electric stimuli have successfully been utilized to trigger the release of molecules via conductive polymeric bulk materials or implantable electronic delivery devices. Drug release systems based on conductive polymers have successfully been utilized, as they offer the possibility of drug administration through electrical stimulation. Ge *et al.*^178^ designed an electric field responsive drug delivery system using nanoparticles of the conductive polymer polypyrrole. Polypyrrole nanoparticles serve as a drug reservoir for electric field triggered release when they are embedded in biocompatible and biodegradable hydrogels of poly[(d,l-lactic acid)-co-(glycolic acid)]-b-poly(ethylene oxide)-b-poly-[(d,l-lactic acid)-co-(glycolic acid)] (PLGA-PEG-PLGA) (Fig. 7a). This gel is injectable (solution at low temperature but converts into a gel at body temperature) and upon application of an external DC electric field, it releases the drug from the nanogel, allowing the drug to diffuse into the surroundings from the hydrogel. Each electric stimulus releases ~25 ng of drug into the solution (Fig. 7b) with minimal release in the absence of an electric field, indicating undesired release from the hydrogel. This type of delivery system has great advantages over conventional sustained drug release because the released dose of this drug can be roughly controlled by either the strength or the duration of the electric field. Electrically controlled drug delivery has been demonstrated by Weaver *et al*.^179^, who used a graphene oxide composite with a polypyrrole scaffold that had a linear release profile under the influence of voltage stimulation, and dosages were adjusted by altering the magnitude of the stimulation, proving on-demand drug delivery. Carbon nanotubes (CNTs) can act as drug nanoreservoirs by holding drug molecules within their inner cavity, releasing them in bioactive form under electrical stimulations^180^. A polypyrrole coating over CNT drug nanoreservoirs seals the ends of the CNTs, effectively loading the drug, which allows electrical triggering to release the drug with the application of voltage^181^. A dual stimuli (electric field and pH) responsive system of chitosan–gold nanocomposites (CGNC) has been designed for site specific controlled delivery of the anticancer drug 5-FU at the reduced pH of cancer cell environments^182^.

### Thermal treatment for cancer therapy

**Fig. 8:**
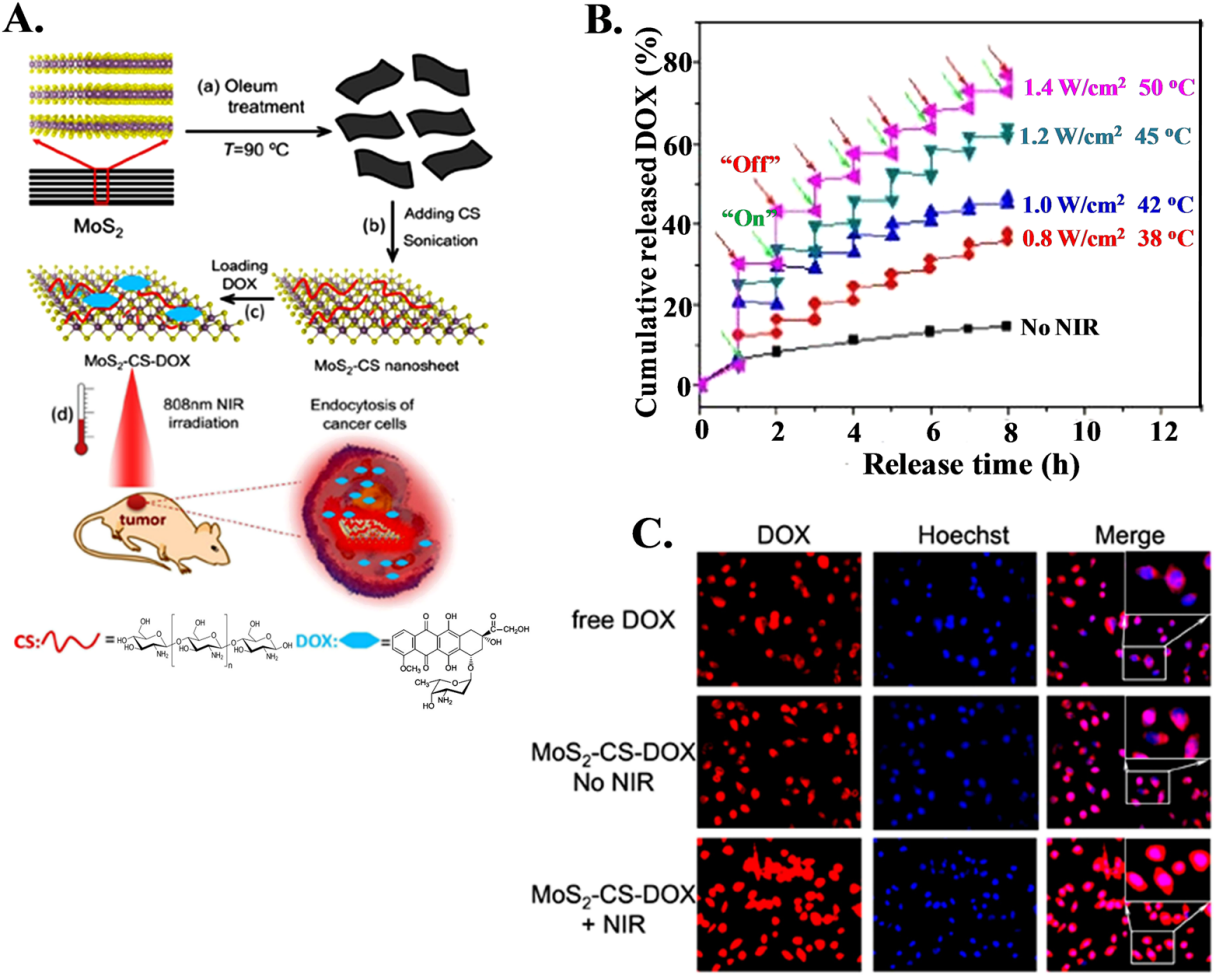
Laser guided control drug delivery using MoS_2_ for cancer treatment. **a** Schematic illustration of high-throughput synthesis of MoS_2_-CS nanosheets as an NIR photothermal-triggered drug delivery system for efficient cancer therapy. (i, ii) Oleum treatment exfoliation process to produce single-layer MoS_2_ nanosheets that are then modified with CS, (iii) DOX loading process, and (iv) NIR photothermal-triggered drug delivery of the MoS_2_ nanosheets to the tumor site. **b** Release profile of DOX in PBS buffer (pH 5.00) in the absence and presence of an 808-nm NIR laser. **c** Fluorescence images of KB cells treated with free DOX, MoS_2_-CS-DOX, and MoS_2_-CS-DOX under 808-nm NIR irradiation (inset: high magnification of the rectangle area)^181^

Photodynamic therapy is an advanced approach that offers control of drug delivery through the use of an external photon source to provide active therapeutic release to a targeted area. Chitosan-functionalized MoS_2_ (MoS_2_-CS) nanosheets can act as a chemotherapeutic drug nanocarrier for near-infrared (NIR) photothermal-triggered drug delivery systems, facilitating the combination of chemotherapy and photothermal treatment for cancer therapy^183^. The synthesis procedure of single-layer MoS_2_ nanosheets and NIR-triggered drug release from MoS_2_ nanocarriers for cancer therapy are shown in Fig. 8a. Drug release profiles show a sharp increase upon irradiation with NIR laser followed by power-dependent release and show nonsignificant release in the absence of irradiation (Fig. 8b). MoS_2_-CS plays an important role in regulating the release of DOX molecules and enhances their nuclear accumulation under NIR irradiation (Fig. 8c). Effective treatment of pancreatic cancer in vivo under NIR irradiation has been carried out, confirming the synergistic efficacy of hyperthermia and chemotherapy. This kind of nanocarrier offers a new possibility for better ‘on-demand’ drug delivery systems that can enhance antitumor efficacy. Dual-in-dual synergistic therapy based on the use of dual anticancer drug-loaded graphene oxide (GO) stabilized with poloxamer 188 has been developed to generate heat and deliver drugs to kill cancer cells under near-infrared (NIR) laser irradiation^184^. Dual drug (doxorubicin and irinotecan)-loaded GO (GO-DI) in combination with laser irradiation caused higher cytotoxicity than that caused by the administration of a free single drug or a combination of drugs and blank GO in various cancer cells, especially in MDA-MB-231 resistant breast cancer cells, suggesting that GO-DI is a powerful tool for drug delivery and can achieve improved therapeutic efficacy and overcome drug resistance in combined chemophotothermal therapy. A photoactivatable *o*-nitrobenzyl (ONB) derivative of 5-fluorouracil (5-FU) attached to the surface of upconverting nanoparticles served as a photocaging nanocarrier that absorbed NIR radiation with upconversion in the UV range, which triggers cleavage of the bonds between ONB-FU at the nanoparticle interface to release chemotherapeutic 5-fluorouracil (5-FU)^185^.

The efficiency of triggered release is sufficiently high (77%) for the total ONB−FU conjugate, whereas the rate of drug release can be tuned with laser power output. The development of this type of UCNP provides a valuable platform for targeted chemotherapy. Thermoresponsive micelles using an amphiphilic diblock copolymer, poly{γ-2-[2-(2-methoxyethoxy)-ethoxy]ethoxy-ε-caprolactone}-b-poly(γ-octyloxy-ε-caprolactone), display a low critical solution temperature (LCST) of 38 °C and can release the therapeutic agent in a controlled fashion^186^. When the anticancer drug doxorubicin is loaded into the micelle, the micelles exhibit statistically higher cytotoxicity against MCF-7 cells at temperatures above the LCST. β-cyclodextrin-poly(*N*-isopropylacrylamide) star polymer is able to form a supramolecular self-assembled inclusion complex with PTX via host–guest interactions at room temperature, which is below the lower critical solution temperature of the star polymer and significantly improves the solubilization of PTX^187^. Phase transitions of poly(*N*-isopropylacrylamide) segments at body temperature (above LCST) induce the formation of nanoparticles, which greatly enhance cellular uptake of the polymer–drug complex, resulting in efficient thermoresponsive delivery of PTX. Dual pH/light-responsive crosslinked polymeric micelles (CPM), prepared by the self-assembly of amphiphilic glycol chitosan-*o*-nitrobenzyl succinate conjugates (GC-NBSCs) and then crosslinked using glutaraldehyde (GA), are used as a drug carrier that can release drugs quickly at low pH under light irradiation^188^. Thus, GC-NBSC CPMs provide a favorable platform to construct dual pH/light-responsive smart drug delivery systems (DDS) for cancer therapy. Biodegradable plasmon resonant liposome gold nanoparticles, which are synthesized using 1,2-distearoyl-sn-glycero-3-phosphocholine (DSPC)-cholesterol coating with gold nanoparticles, are capable of killing cancer cells through photothermal therapy.

### Future challenges in cancer therapy

Novel drug delivery systems promise a bright future for cancer treatment in the next decade or so; they might become major arsenal for safer and more efficient treatments by ensuring proper drug localization at the site of action in a controlled manner. The enhanced therapeutic efficacy of targeted nanocarriers has been established in cancer treatment using multiple animal models that target tumors and deliver drugs for targeted radiotherapy, imaging-guided radiotherapy and precision medicine^189,190^. Although major advances have been made by current drug delivery systems in the treatment of most cancers, much work lies ahead to monitor the mortality rate due to cancer. Most of these carriers have been designed and tested in small animal models, achieving great therapeutic results; however, the translation of animal results into clinical success has been limited. More clinical data are needed to fully understand the advantages and disadvantages of these vehicles. Now, we have entered into an era of molecular targeting of cancer that may further improve the chemotherapeutic index by detecting malignant cells (active targeting moiety), tracking their location in the body (real-time in vivo imaging), killing cancer cells while producing minimal adverse side effects by sparing normal cells (active targeting and controlled drug release or hyperthermia ablation) and monitoring the study in real-time. Ion beam therapy seems to be a promising tool for oncologists to treat cancer in near the future instead of high-risk surgery, widespread damage from other forms of radiation therapy, such as X-rays, or collateral damage induced by chemotherapeutic drugs. Classic radiation treatment involves mainly X-rays, which lose energy all along their path through the body and thereby damage healthy cells in their path. The beams of protons or heavier ions, such as carbon and neon, can be accelerated precisely with calculated energy to accurately target tumor cells, sparing healthy tissue above and below the targeted site. The main advantage of employing ion beam radiation for cancer treatment is that it has the potential to precisely target any type or form of tumor, which may be very small or large and may be dangerously shaped or positioned surrounding the spinal cord, in the center of the brain or close to the optic nerves. Even though proton therapy is commonly used at present, heavier carbon ions deposit more energy in tumor tissues. Therefore, carbon or other heavier elements are considerably more destructive towards the tumor, and hence, they require a fewer number of doses for treatment. For example, liver cancer requires 30 days of treatment using proton therapy, whereas only just four days of treatment is sufficient for carbon therapy. Carbon therapy provides the highest linear energy transfer (LET) of any currently available form of clinical radiation. This high energy irradiation to tumor cells results in the destruction of most double-stranded DNA; this extensive destruction is very difficult for other conventional radiation therapies to accomplish, as they predominantly break single-stranded DNA. Recent technological advancements in the fields of accelerator engineering, beam delivery, treatment planning, and tumor visualization have transferred ion beam therapy from physics laboratories to clinics.

## Conclusions

This review has summarized a variety of materials that are either being used or have the potential to be used as drug delivery vehicles for the treatment of cancer. Their unique attributes have allowed clinicians to offer them as new treatments (monotherapy) or as adjuncts to existing treatments (combined therapy) to improve therapeutic effectiveness. Although some of these materials have not been successful upon their clinical translation, several new and promising materials that are currently under development show great promise, thereby providing hope for new treatment options in the near future.
